# Contribution of Beef to Key Nutrient Intakes and Nutrient Adequacy in Pregnant and Lactating Women: NHANES 2011–2018 Analysis

**DOI:** 10.3390/nu16070981

**Published:** 2024-03-28

**Authors:** Sanjiv Agarwal, Victor L. Fulgoni

**Affiliations:** 1 NutriScience, LLC, East Norriton, PA 19403, USA; 2 Nutrition Impact, LLC, Battle Creek, MI 49014, USA; vic3rd@aol.com

**Keywords:** fresh beef, ground beef, processed beef, protein, B-vitamins, iron, zinc, choline

## Abstract

Beef is an important source of high-quality protein and several micronutrients, including iron, zinc, and B-vitamins. The objective was to assess the association of beef intake with nutrient intake and adequacy among pregnant and lactating women using 24-h dietary recall data. Usual intakes from foods were determined with the National Cancer Institute (NCI) method and % population below Estimated Average Requirement (EAR) or above Adequate Intake (AI) were estimated. A high proportion of pregnant and lactating women had inadequate intakes for vitamin D (94%), vitamin E (82%), vitamin C (52%), and vitamin A (50%), magnesium (35%), folate (31%), zinc (25%), and vitamin B_6_ (22%); only 4% and 35% met AI for choline and potassium, respectively. About 67% of pregnant and lactating women were beef consumers, consuming 49 g beef/day. Beef consumers had higher intakes (*p* < 0.05) of energy, protein, calcium, iron, phosphorus, selenium, sodium, zinc, thiamin, riboflavin, and niacin, and a higher proportion (*p* < 0.05) met nutrient recommendations for protein, calcium, iron, zinc, thiamin, riboflavin, niacin, vitamin B_6_, and vitamin B_12_ compared to non-consumers. In conclusion, pregnant and lactating women generally have inadequate nutrient intakes from their diets. Beef consumers have higher intakes and adequacy for certain nutrients, many of which are inherently available in beef or in foods eaten with beef.

## 1. Introduction

Pregnancy and lactation are important stages of life during which proper nutrition plays an important role to support the health of the mother and child. The importance of adequate nutrition during pregnancy and lactation for both maternal and child health outcomes is well recognized [1,2,3,4,5]. Nutrition and lifestyle during these stages of life have been shown to induce life-long effects on health of the child, including the risk of diseases. This has been referred to as “early metabolic programming or developmental origins of health and disease” [6,7,8]. Women undergo several hormonal, metabolic, and physiological changes during pregnancy; energy and nutrient requirements, especially for folate, iron, iodine, and copper, are increased throughout both pregnancy and lactation to support the normal development and health of the fetus and infant [9,10]. As such, increased intakes of nutrient dense foods are recommended [1,2]. However, to date, only limited data are available on nutrient intakes and nutrient adequacy estimates in pregnant women and almost no data are available for lactating women in the US. Bailey et al. [11], using National Health and Nutrition Examination Survey (NHANES) 2001–2014 data, reported that even with the use of dietary supplements a significant proportion of pregnant and non-lactating women are not meeting recommendations for multiple vitamins and minerals, including vitamin A, folate, vitamin B_6_, vitamin C, vitamin D, vitamin E, vitamin K, choline, calcium, iron, magnesium, potassium and zinc. Crawford et al. [12], using baseline dietary intake data from a multisite clinical trial of docosahexaenoic acid (DHA) supplementation, also reported that dietary intakes of folate, vitamin D, choline, zinc, vitamin E, iron, magnesium, and potassium were below the Adequate Intake (AI) or the Estimated Average Requirement (EAR) for 30–91% of the cohort of pregnant women participants. Another more recent analysis of NHANES 2003–2016 also reported that pregnant women have inadequate intakes of vitamin A, vitamin D, calcium, magnesium, and zinc [13].

In the American diet, beef is a staple food; it provides high-quality animal protein and several key micronutrients, including highly bioavailable iron, zinc, and B vitamins [14,15,16,17]. Intake of lean beef was shown to contribute to energy and key nutrients intakes among US adults in an earlier analysis of NHANES 1999–2004 [18] and consumers of beef with the highest lean meat and lowest fat content had higher intakes of protein as well as B vitamins, zinc, iron, and potassium than non-consumers [19]. In another analysis of NHANES 2015–2016, fresh and lean beef intake was associated with higher intake of protein, sodium, choline, iron, selenium, zinc, phosphorus, and B vitamins along with energy and fat in adults [20]. Using NHANES 2011–2018 data, we recently reported that intake of beef contributed significant amount of protein, B vitamins, zinc, and iron in the diet of American adults excluding pregnant and lactating women [21].

We hypothesize that since pregnant and lactating women have increased needs for but inadequate intake of nutrients, intake of beef as a rich source of protein and other nutrients would be associated with improved nutrient intakes and nutrient adequacy. Therefore, the objective of the present research was to assess the association of intake of beef (including fresh, ground, and processed beef) with meeting nutrient recommendations in pregnant and lactating women using the National Health and Nutrition Examination Survey (NHANES) 2011–2018.

## 2. Materials and Methods

### 2.1. Database and Participants

Data from pregnant and lactating female participants in the NHANES 2011–2012, 2013–2014, 2015–2016, and 2017–2018 were combined for the analyses [22]. NHANES is a continuous cross-sectional survey conducted by National Center for Health Statistics (NCHS) of Centers for Disease Control and Prevention (CDC) and is based on a complex stratified multistage cluster sampling probability design to provide a nationally representative population to monitor food and nutrient intake and the health status of the US population. Self-reported pregnancy and lactation status were obtained from the demographic files. Participants were interviewed in their homes for demographic, socioeconomic, dietary (24-h dietary recall), and general health information, followed by a comprehensive health examination conducted in a mobile examination center. A detailed description of the subject recruitment, survey design, and data collection procedures is available online [22]. The total number of pregnant and lactating female subjects were 319 (193 pregnant and 126 lactating women) after exclusion for subjects with missing day 1 or day 2 dietary data. The NHANES research protocol was approved by the NCHS Research Ethics Review Board and all participants provided signed written informed consent. NHANES has stringent consent protocols and procedures to ensure confidentiality and protection from identification. The present study was a secondary data analysis that lacked personal identifiers and was therefore exempt from additional approvals by institutional review boards. All data obtained in this study are publicly available at [http://www.cdc.gov/nchs/nhanes/ (accessed on 10 December 2023)].

### 2.2. Dietary Intake

Dietary intake (from food only) data were obtained from 24-h dietary recall interviews that were administered using an automated, multiple-pass (AMPM) method [23]. As part of the NHANES examination, details of all foods and beverages consumed by respondents in the previous 24-h period (midnight to midnight) were collected in person by a trained dietary interviewer, followed by a second dietary recall phone interview for most subjects 3 to 10 days after the first dietary interview. Nutrient intakes from foods only were determined using the NHANES cycle specific the USDA Food and Nutrient Database for Dietary Studies (FNDDS) for each NHANES cycle [24].

### 2.3. Beef Intake

Beef intakes were assessed by methods previously reported [21] and briefly summarized here. First, the amount of beef contained in survey foods was determined using the FNDDS food codes. The FNDDS food codes for beef used as “ingredients” of the survey foods were identified and recipe calculations were performed using the survey-specific USDA Food Patterns Equivalents (FPED) database, which also includes the Food Patterns Equivalents Ingredient Database (FPID) [25]. The proportion of beef in the ingredient was determined by examining the FPID descriptions: 100% if entirely beef, and 50% or 33% if the description indicated one or two other meat types in addition to beef, respectively. For some FNDDS food codes that contained ingredients with missing FPID or food codes for beef, the food code ingredient profile was modified either by using food code from another NHANES cycle or by using another ingredient code with a similar description. Fresh beef and processed beef were defined by using “pf_meat” and “pf_curedmeat” components, respectively, and ground beef was determined based on the ingredient description of beef containing ingredients (i.e., ground beef or similar term) [25]. All beef included fresh, ground, and processed beef and consumer status was based on any consumption of beef on either of two days observed intake.

### 2.4. Statistics

All analyses were performed using SAS 9.4 (SAS Institute, Cary, NC, USA) software after adjusting the data for the complex sampling design of NHANES, using appropriate survey weights, strata, and primary sampling units. Two-day dietary weights were used in all intake analysis. The distributions of usual nutrient intakes were estimated by using the National Cancer Institute (NCI) method [26] and the percentage of the population below the Estimated Average Requirement (EAR) or above the Adequate Intake (AI) using the cut-point method except for iron where we used the probability method [27]. Data are presented as mean ± standard error; t-tests were used to assess differences between non-consumers and consumers.

## 3. Results

The demographics of pregnant and lactating women participants of NHANES 2011–2018 is presented in Table 1. The mean age of pregnant and lactating women participants was about 30 years. More than half of pregnant and lactating women participants were non-Hispanic white and a fifth were Hispanic. More than half of pregnant and lactating women participants had a household poverty income ratio (PIR) above 1.85 and one third had a PIR below 1.35. About 30% of participants had a high school education or less and about a 40% of participants had at least a Bachelor’s degree. About half of participants were moderately active, a third of participants were vigorously active, and about a fifth of participants were sedentary. About three quarters of participants never smoked (Table 1).

About 67% of pregnant and lactating women were consumers of beef, with a mean intake of 49.3 ± 3.5 g/day among consumers; mean per capita intake was 33.3 ± 3.2 g/day. Consumers of beef were slightly younger, engaged in moderate exercise, and a lower percentage had a Bachelor’s degree or more education, engaged in vigorous exercise, and were non-smokers than non-consumers (Table 1).

The current (2011–2018) usual intakes of key nutrients and the % of the pregnant and lactating women population meeting nutrient recommendations among are presented in Table 2. Prevalence of nutrient inadequacy was high among pregnant and lactating women with over three quarters having intakes being below EAR for vitamin D (94%) and vitamin E (82%); about half were below EAR for vitamin C (52%) and vitamin A (50%); about a third had intakes below EAR for magnesium (35%) and folate (31%); about a quarter had intakes below EAR for vitamin B_6_ (22%) and zinc (25%); and more than a tenth had intakes below EAR for calcium (12%) and copper (12%). Additionally, only about 4% and 35% of pregnant and lactating women were meeting AI for choline and potassium, respectively, while all pregnant and lactating women exceeded the AI for sodium.

The current nutrient intake and % of the population meeting nutrient recommendations among pregnant and lactating women beef consumers and non-consumers are presented in Table 3. Consumers of beef had higher usual intakes of energy (+17%), protein (+21%), calcium (+17%), iron (+21%), phosphorus (+11%), selenium (+19%), sodium (+28%), zinc (+41%), thiamin (+25%), riboflavin (+16%), and niacin (+25%) compared to non-consumers. A higher proportion of beef consumers met the nutrient recommendations for protein (20% units), calcium (28% units), iron (9% units), zinc (59% units), thiamin (23% units), riboflavin (14% units), niacin (9% units), vitamin B_6_ (20% units), and vitamin B12 (22% units) compared to non-consumers. However, a lower proportion (−16% units) of beef consumers compared to non-consumers met nutrient recommendation for vitamin D (Table 3).

About 59%, 42%, and 23% of pregnant and lactating women were consumers of fresh beef, ground beef and processed beef, respectively, with a mean intake of 50.0 ± 3.6, 38.6 ± 2.8, and 16.2 ± 5.0 g/day among consumers. Mean per capita intake of fresh beef, ground beef, and processed beef were 29.6 ± 3.1, 16.3 ± 1.9, and 3.67 ± 1.35 g/day, respectively. Percentage of the population meeting nutrient recommendations among consumers of different beef types are presented in Table 4. A higher proportion of consumers of fresh beef met the nutrient recommendations for calcium (25% units), iron (7% units), zinc (52% units), thiamin (16% units), and vitamin B_12_ (14% units) compared to non-consumers. Similarly, a higher proportion consumers of ground beef met the nutrient recommendations for protein (10% units), calcium (27% units), iron (6% units), zinc (41% units), thiamin (14% units), riboflavin (8% units), niacin (4% units), folate (29% units), and vitamin B_12_ (10% units) than non-consumers. Consumers of processed beef also had a higher proportion of consumers that met the nutrient recommendations for protein (11% units), calcium (20% units), copper (14% units), iron (7% units), zinc (35% units), thiamin (9% units), riboflavin (9% units), niacin (5% units), folate (25% units), and vitamin B_12_ (8% units) than non-consumers. However, a higher proportion of consumers of fresh beef and ground beef failed to meet nutrient recommendations for vitamin D (13% units) and had a lower proportion meeting the AI for choline (6% units) compared to their respective non-consumers.

## 4. Discussion

To the best of our knowledge, this is the first report to investigate the association between beef intake and meeting nutrient recommendations in American pregnant and lactating women. The results of this analysis of cross-sectional data from NHANES 2011–2018 indicate that pregnant and lactating women consumers of beef have higher intakes and lower prevalence of inadequacies of several key micronutrients, including many under-consumed nutrients and nutrients of concern, compared to non-consumers of beef. Interestingly, the results for most nutrients are similar for consumers of different beef types.

Dietary Guidelines for Americans 2020–2025 recognized the criticality of maternal and postnatal nutrition for lifelong health and well-being and, for the first time, provided recommended dietary patterns for women during pregnancy and lactation [1]. However, only limited US data are available on the dietary intakes of pregnant and lactating women. In the present cross-sectional analysis on dietary intakes of 319 pregnant and lactating women who participated in NHANES 2011–2018 (representing about 4.2 million women), we find a high prevalence of micronutrient inadequacy (estimated as large % population below EAR), including inadequate intakes of vitamin D, vitamin E, vitamin C, and vitamin A among the majority (over 50% population) of pregnant and lactating women and inadequate intakes of magnesium, folate, vitamin B6, zinc, calcium, and copper among a significant proportion (over 10%) of pregnant and lactating women. Intakes of potassium and choline were also lower than recommended levels, with less than 30% of the population meeting the AI for potassium and less than 10% meeting the AI for choline. A similar high prevalence of nutritional inadequacies for several vitamins and minerals among pregnant women were also reported earlier [11,12,13].

In the present analysis, the pregnant and lactating women consumers of beef had higher intakes of energy, protein, calcium, iron, phosphorus, selenium, sodium, zinc, thiamin, riboflavin, and niacin, and lower prevalence of inadequacy for protein, calcium, iron, zinc, thiamin, riboflavin, niacin, vitamin B_6_, and vitamin B_12_ compared to non-consumers. Interestingly the prevalence of nutritional inadequacy for protein, niacin, thiamin, and vitamin B_12_ were close to zero among beef consumers. To put these results into perspective, a sample size of 319 represented 4.2 million women, of which 1.4 million (sample size 100) were non-consumers and 2.9 million (sample size 219) were consumers of beef. The results of a decrease in percentage of pregnant and lactating women population below the EAR for zinc from 64.6% in non-consumers to 5.29% in consumers of beef suggest that about 0.83 million pregnant and lactating women non-consumers ((64.6–5.29%) × 1.4 million non-consumers) would no longer be below the EAR for zinc if they consumed a food pattern similar to beef consumers and chose to incorporate beef into their diet. Similarly, by multiplying the difference in consumer and non-consumers for nutrient adequacy with the population of non-consumers, we also estimated that about 0.28, 0.39, 0.13, and 0.27 million pregnant and lactating women non-consumers would no longer be below the EAR for protein, calcium, iron, and vitamin B_6_, respectively, if they chose to incorporate beef into their diet and consumed a food pattern similar to beef consumers in this study.

Interestingly, while consumers of beef had significantly higher intakes of phosphorus and selenium compared to non-consumers, the difference in the prevalence of inadequacy for these nutrients did not reach statistical significance. Similarly, consumers of beef had a lower prevalence of inadequacy of vitamin B_6_ and vitamin B_12_ and higher inadequacy for vitamin D compared to non-consumers, but the differences in their intakes did not reach statistical significance.

To the best of our knowledge, this is also the first study that examined the intake of different types of beef, including fresh, ground, and processed, in relation to their contribution to the percentage meeting nutrient recommendations in US pregnant and lactating women. We recently reported that intake of different types of beef, including lean fresh, ground, and processed, contribute to energy, protein, and multiple key micronutrients in the diet of American adults, excluding analysis of pregnant and lactating women [21]. In the present analysis, consumers of different beef types also had similar generally lower prevalence of inadequacy for calcium, iron, zinc, thiamin, riboflavin, niacin, and vitamin B_12_ compared to their respective non-consumers. However, differences in adequacies in protein, riboflavin, and niacin did not reach statistical significance in consumers of fresh beef.

It is also of interest to note that while the beef intake was associated with higher beef specific nutrients such as iron, B-vitamins, and zinc, consumers of beef also had higher intakes and lower prevalence of inadequacy of calcium. Since beef is generally not considered a source of calcium and contributes only small amount in the diet [21], the higher intakes and lower inadequacy of calcium could be due to overall dietary pattern food group intakes among beef consumers; differences likely include more dairy (milk, yogurt, and cheese) consumption. However, consumers of beef had also had higher prevalence of inadequacy for vitamin D, and beef intake was not associated with changes in nutrient inadequacy for vitamins A, C, and E where a large percentage of both non-consumers and consumers of beef had intake below the EAR.

This study has several strengths, including the use of NHANES, a large nationally representative dataset and the NCI method to assess usual intake and nutritional inadequacy. On the other hand, this study has several limitations. Although we used several cycles of NHANES and combined pregnant and lactating women to increase the sample size, it was still relatively small. The cross-sectional analyses of NHANES cannot be used to assess causal relationships. While this study utilized 24-h dietary recalls collected on two different days, it is possible participants consumed beef on days other than those reported, which would result in underestimating the beef intake. The self-reported dietary recalls are based on memory and are known to be subject to reporting bias [28]. It is likely that the associations of beef intake with nutrient intakes and % below the EAR/above the AI as noted in the present analysis may (at least in some part) also be due to the differences in other dietary constituents of beef consumers and non-consumers. Additionally, nutrient intakes were estimated only from foods and intakes from dietary supplements were not included.

## 5. Conclusions

The results show that beef intake was associated with improved nutrient intake and meeting nutrient recommendations in US pregnant and lactating women for certain key nutrients. It is therefore likely that beef may play a critical role in reducing the incidence of under-nutrition in this population. Beef’s nutrient density (e.g., protein, iron, phosphorus, zinc, vitamin B_12_, choline) [14,15,16,17] makes it a particularly important food in the diets of pregnant and lactating women. The results suggest the development of dietary recommendations to reduce or limit beef for whatever reason should take into consideration whether other foods in the dietary recommendation provide the essential nutrients that could be expected to be provided by beef (e.g., protein, iron, phosphorus, zinc, vitamin B_12_, choline) in pregnant and lactating women. Future studies are needed to examine the long-term impact of beef consumption on nutrient intake and diet quality in pregnant and lactating women.

## Figures and Tables

**Table 1:** Pregnant and lactating women demographics associated with beef consumption, NHANES 2011–2018.

	Total Population	Beef		
		Non-Consumers	Consumers	*p* Value
Sample n	319	100	219	
Population N	4,234,347	1,378,135	2,856,212	
Age (mean)	29.7 ± 0.6	31.0 ± 0.9	29.0 ± 0.7	0.0442
Ethnicity				
Hispanic (%)	21.3 ± 3.3	22.2 ± 6.5	20.8 ± 4.6	0.8769
Non-Hispanic White (%)	53.3 ± 5.7	52.0 ± 8.9	54.0 ± 6.6	0.8419
Non-Hispanic Black (%)	15.5 ± 2.9	12.1 ± 3.3	17.1 ± 3.3	0.1549
Non-Hispanic Asian (%)	6.77 ± 1.40	11.0 ± 3.4	4.73 ± 1.23	0.0843
Other (%)	3.12 ± 0.92	2.66 ± 1.75	3.35 ± 0.99	0.7248
Poverty Income Ratio				
<1.35 (%)	33.3 ± 3.2	30.8 ± 4.9	34.5 ± 4.4	0.6009
1.35 ≤ 1.85 (%)	6.92 ± 1.75	4.72 ± 2.12	7.98 ± 2.13	0.2144
>1.85 (%)	59.8 ± 3.1	64.4 ± 5.0	57.5 ± 4.2	0.3243
Education				
≤High school degree (%)	29.8 ± 4.0	20.5 ± 5.7	34.3 ± 4.9	0.0729
Some post high school education (%)	30.0 ± 3.1	27.3 ± 5.2	31.4 ± 3.8	0.5259
≥Bachelor’s degree(%)	40.2 ± 3.8	52.2 ± 5.8	34.3 ± 4.6	0.0218
Physical Activity				
Sedentary (%)	18.9 ± 2.9	20.3 ± 4.5	18.3 ± 3.3	0.6819
Moderate (%)	47.3 ± 3.1	33.8 ± 5.1	53.9 ± 3.6	0.0013
Vigorous (%)	33.7 ± 3.0	45.9 ± 5.7	27.9 ± 3.1	0.0056
Smoking Never (%)	76.4 ± 3.3	88.3 ± 4.2	70.7 ± 3.8	0.0015
Smoking Current (%)	6.81 ± 2.05	3.05 ± 1.70	8.62 ± 2.72	0.0691

Consumer status based on two days 24-h dietary recall data from NHANES 2011–2018. Data is presented as Mean ± Standard Error.

**Table 2:** Usual intakes of nutrients and percentage of the pregnant and lactating women meeting nutrient recommendations (n = 319).

	Usual Intake	% Below EAR or Above AI
Energy (kcal)	2175 ± 51	
EAR Nutrients		
Protein (g)	82.9 ± 2.0	6.74 ± 1.92
Calcium (mg)	1116 ± 32	12.5 ± 4.0
Copper (mg)	1.37 ± 0.06	11.9 ± 3.2
Iron (mg)	16.6 ± 0.6	7.31 ± 1.36
Magnesium (mg)	318 ± 11	34.5 ± 4.8
Phosphorus (mg)	1443 ± 30	0.20 ± 0.30
Selenium (µg)	118 ± 4	0.96 ± 0.60
Zinc (mg)	11.7 ± 0.4	24.6 ± 5.4
Vitamin A, RAE (µg)	691 ± 32	49.6 ± 5.4
Thiamin (mg)	1.81 ± 0.07	7.45 ± 2.61
Niacin (mg)	26.5 ± 0.9	3.24 ± 0.85
Riboflavin (mg)	2.16 ± 0.05	6.28 ± 1.60
Folate, DFE (µg)	648 ± 34	30.6 ± 5.4
Vitamin B_6_ (mg)	2.19 ± 0.09	22.1 ± 4.3
Vitamin B_12_ (µg)	5.17 ± 0.30	7.18 ± 1.97
Vitamin C (mg)	90.6 ± 5.2	52.3 ± 3.7
Vitamin D (µg)	5.31 ± 0.43	93.6 ± 2.5
Vitamin E, ATE (mg)	10.1 ± 0.6	82.0 ± 5.0
AI Nutrients		
Potassium (mg)	2627 ± 66	35.4 ± 4.1
Sodium (mg)	3692 ± 129	99.9 ± 0.2
Choline (mg)	314 ± 9	3.57 ± 1.31

Two days 24-h dietary recall data from NHANES 2011–2018. Data is presented as Mean ± Standard Error. AI: adequate intake; ATE: alpha tocopherol equivalents; EAR: estimated average intake; DFE: dietary folate equivalents; RAE: retinol activity equivalents.

**Table 3:** Usual intakes of nutrients and percentage of the population meeting nutrient recommendations among pregnant and lactating women non-consumers and consumers of beef.

	Usual Intakes			% Meeting Recommendations		
	Beef Non-Consumers	Beef Consumers	*p* Value	Beef Non-Consumers	Beef Consumers	*p* Value
Energy (kcal)	1949 ± 99	2284 ± 71	0.0059			
	EAR Nutrients			% below EAR		
Protein (g)	72.6 ± 3.3	87.6 ± 2.9	0.0006	20.4 ± 5.9	<1.00	0.0007
Calcium (mg)	996 ± 49	1170 ± 44	0.0087	31.8 ± 5.3	3.71 ± 5.39	0.0002
Copper (mg)	1.32 ± 0.12	1.40 ± 0.07	0.5571	17.0 ± 7.3	8.64 ± 3.09	0.2878
Iron (mg)	14.5 ± 1.2	17.6 ± 0.8	0.0294	13.3 ± 2.9	4.16 ± 1.51	0.0046
Magnesium (mg)	313 ± 19	322 ± 13	0.7113	36.7 ± 9.8	32.5 ± 5.2	0.7000
Phosphorus (mg)	1346 ± 56	1491 ± 45	0.0443	<1.00	<1.00	0.3835
Selenium (µg)	104 ± 5	124 ± 6	0.0052	2.67 ± 1.94	<1.00	0.1694
Zinc (mg)	9.13 ± 0.40	12.9 ± 0.5	<0.0001	64.6 ± 5.6	5.29 ± 6.63	<0.0001
Vitamin A, RAE (µg)	720 ± 45	686 ± 44	0.5933	54.4 ± 4.6	45.7 ± 7.7	0.3337
Thiamin (mg)	1.54 ± 0.06	1.93 ± 0.10	0.0005	23.4 ± 7.0	<1.00	0.0011
Riboflavin (mg)	1.95 ± 0.10	2.26 ± 0.09	0.0185	15.7 ± 4.4	1.43 ± 1.21	0.0015
Niacin (mg)	22.7 ± 1.6	28.4 ± 1.2	0.0054	9.49 ± 2.67	<1.00	0.0005
Folate, DFE (µg)	583 ± 65	681 ± 45	0.2147	41.2 ± 10.1	25.1 ± 5.9	0.1656
Vitamin B_6_ (mg)	2.00 ± 0.14	2.28 ± 0.13	0.1521	35.5 ± 6.3	15.9 ± 6.2	0.0261
Vitamin B_12_ (µg)	4.27 ± 0.59	5.56 ± 0.33	0.0564	21.6 ± 6.0	<1.00	0.0003
Vitamin C (mg)	94.9 ± 7.7	88.6 ± 7.2	0.5495	51.6 ± 4.6	52.7 ± 5.4	0.8793
Vitamin D (µg)	6.29 ± 1.13	4.77 ± 0.33	0.1970	82.8 ± 7.1	99.1 ± 1.1	0.0235
Vitamin E, ATE (mg)	10.3 ± 1.2	9.98 ± 0.62	0.7960	80.8 ± 7.4	82.1 ± 5.8	0.8913
	AI Nutrients			% Above AI		
Potassium (mg)	2473 ± 114	2701 ± 92	0.1203	29.4 ± 5.0	37.8 ± 5.9	0.2796
Sodium (mg)	3111 ± 134	3985 ± 180	0.0001	99.7 ± 0.5	>99.9	0.5247
Choline (mg)	296 ± 17	322 ± 13	0.2176	7.72 ± 2.31	1.90 ± 2.04	0.0592

Consumer status based on two days 24-h dietary recall data from NHANES 2011–2018. Data is presented as Mean ± Standard Error. AI: adequate intake; ATE: alpha tocopherol equivalents; EAR: estimated average intake; DFE: dietary folate equivalents; RAE: retinol activity equivalents.

**Table 4:** Percentage of the population meeting nutrient recommendations among pregnant and lactating women non-consumers and consumers of different beef types.

	Fresh Beef		Ground Beef		Processed Beef	
	Non-Consumers (n = 129)	Consumers (n = 190)	Non-Consumers (n = 185)	Consumers (n = 134)	Non-Consumers (n = 243)	Consumers (n = 76)
% below Estimated Average Requirement (EAR)						
Protein	10.9 ± 5.9	0.82 ± 2.69	9.93 ± 4.77	<1.00 ^#^	11.0 ± 3.0	<1.00 *
Calcium	28.2 ± 6.7	2.79 ± 5.17 *	27.2 ± 4.5	<1.00 *	19.8 ± 3.5	<1.00 *
Copper	15.1 ± 5.5	8.64 ± 2.98	14.2 ± 3.8	5.76 ± 3.15	13.8 ± 2.6	<1.00 ^#^
Iron	11.3 ± 3.1	4.48 ± 1.52 ^#^	9.36 ± 1.77	2.88 ± 1.90 ^#^	8.74 ± 1.34	1.57 ± 2.54 ^#^
Magnesium	37.1 ± 10.4	32.9 ± 5.3	33.6 ± 6.7	34.9 ± 8.6	35.8 ± 4.4	23.8 ± 22.6
Phosphorus	<1.00	<1.00	<1.00	<1.00	<1.00	<1.00
Selenium	<1.00	<1.00	<1.00	<1.00	<1.00	<1.00
Zinc	58.0 ± 9.7	6.35 ± 7.40 *	42.4 ± 7.6	1.53 ± 9.19 *	35.0 ± 4.8	<1.00 *
Vitamin A (RAE)	57.1 ± 5.0	43.6 ± 8.0	55.2 ± 4.7	40.3 ± 10.4	51.4 ± 4.6	39.8 ± 14.3
Thiamin	16.5 ± 6.2	<1.00 ^#^	14.5 ± 3.9	<1.00 *	9.34 ± 4.64	<1.00 ^#^
Riboflavin	11.3 ± 4.8	2.29 ± 1.62	9.11 ± 2.64	<1.00 *	8.62 ± 2.06	<1.00 *
Niacin	3.13 ± 2.17	<1.00	4.42 ± 2.06	<1.00 ^#^	4.71 ± 1.60	<1.00 *
Folate, DFE	37.4 ± 7.9	25.0 ± 8.2	36.9 ± 5.7	8.32 ± 12.99 ^#^	37.4 ± 5.0	12.1 ± 10.3 ^#^
Vitamin B6	27.3 ± 9.8	20.6 ± 5.2	24.5 ± 5.5	17.5 ± 7.5	27.2 ± 4.4	9.85 ± 11.51
Vitamin B12	14.4 ± 5.4	<1.00 *	10.1 ± 3.5	<1.00 *	8.21 ± 3.61	<1.00 ^#^
Vitamin C	55.0 ± 5.9	52.0 ± 5.5	51.4 ± 5.3	52.3 ± 6.4	49.9 ± 3.6	61.2 ± 12.3
Vitamin D	85.7 ± 6.2	98.7 ± 1.5 ^#^	89.2 ± 4.9	99.3 ± 2.1	92.9 ± 3.7	98.8 ± 2.6
Vitamin E (ATE)	82.9 ± 4.6	80.9 ± 6.2	84.6 ± 3.9	78.5 ± 8.6	80.4 ± 5.2	86.2 ± 5.9
% above Adequate Intake (AI)						
Potassium	26.6 ± 5.6	40.9 ± 6.4	35.5 ± 5.2	34.0 ± 7.2	34.9 ± 3.8	38.6 ± 27.4
Sodium	>99.9	>99.9	99.4 ± 0.6	>99.9	99.6 ± 0.4	>99.9
Choline	6.21 ± 2.16	1.73 ± 1.98	5.58 ± 1.60	<1.00 ^#^	3.56 ± 1.48	1.32 ± 3.27

Consumer status based on two days 24-h dietary recall data from NHANES 2011–2018. Data is presented as Mean ± Standard Error. ^#^, * represent significant differences from non-consumers at *p* < 0.05 and *p* < 0.01, respectively. ATE: alpha tocopherol equivalents; DFE: dietary folate equivalents; RAE: retinol activity equivalents.

## Data Availability

The datasets analyzed in this study are available in the Center for Disease Control and Prevention repository; available online: http://www.cdc.gov/nchs/nhanes/ (accessed on 10 December 2023).
